# The Role of Long-Term Head-Down Bed Rest in Understanding Inter-Individual Variation in Response to the Spaceflight Environment: A Perspective Review

**DOI:** 10.3389/fphys.2021.614619

**Published:** 2021-02-11

**Authors:** Jonathan P. R. Scott, Andreas Kramer, Nora Petersen, David A. Green

**Affiliations:** ^1^Space Medicine Team, ISS Operations and Astronaut Group, Directorate of Human and Robotic Exploration, European Space Agency, Cologne, Germany; ^2^KBR GmbH, Cologne, Germany; ^3^Department of Sport Science, University of Konstanz, Konstanz, Germany; ^4^Centre of Human and Applied Physiology, King’s College London, London, United Kingdom

**Keywords:** microgravity, countermeasure exercise, spaceflight, inter-individual variability, bed rest, musculoskeletal, cardiorespiratory

## Abstract

Exposure to the spaceflight environment results in profound multi-system physiological adaptations in which there appears to be substantial inter-individual variability (IV) between crewmembers. However, performance of countermeasure exercise renders it impossible to separate the effects of the spaceflight environment *alone* from those associated with exercise, whilst differences in exercise programs, spaceflight operations constraints, and environmental factors further complicate the interpretation of IV. In contrast, long-term head-down bed rest (HDBR) studies isolate (by means of a control group) the effects of mechanical unloading from those associated with countermeasures and control many of the factors that may contribute to IV. In this perspective, we review the available evidence of IV in response to the spaceflight environment and discuss factors that complicate its interpretation. We present individual data from two 60-d HDBR studies that demonstrate that, despite the highly standardized experimental conditions, marked quantitative differences still exist in the response of the cardiorespiratory and musculoskeletal systems between individuals. We also discuss the statistical concept of “true” and “false” individual differences and its potential application to HDBR data. We contend that it is currently not possible to evaluate IV in response to the spaceflight environment and countermeasure exercise. However, with highly standardized experimental conditions and the presence of a control group, HDBR is suitable for the investigation of IV in the physiological responses to gravitational unloading and countermeasures. Such investigations may provide valuable insights into the potential role of IV in adaptations to the spaceflight environment and the effectiveness of current and future countermeasures.

## Introduction

Inter-individual variation (IV), where participants display markedly different responses to a standardized intervention, is a recognized phenomenon in clinical and basic research studies (Bouchard and Rankinen, 2001; Timmons, 2010). This variation in responses led, initially, to the adoption of terminology such as “responders” and “non-responders” (Timmons, 2010), which has subsequently evolved into more precise definitions including individuals that “did not respond” (Pickering and Kiely, 2019) or have “low sensitivity” (Booth and Laye, 2010). The careful identification and quantification of IV has important implications, not only the optimization of health interventions, but also determination of pathophysiological processes that can underpin the provision of personalized medicine. Should such IV also exist in response to the spaceflight environment, known to induce multi-system physiological adaptation (Demontis et al., 2017), and the performance of countermeasure (CM) exercise in an attempt to mitigate these adaptations (Loehr et al., 2015), this could have important implications for astronaut health management, particularly during future exploration missions where the operational constraints will be more severe (Scott et al., 2019).

In this Perspective, we review the available evidence of IV in response to the spaceflight environment and discuss biological, operational, and environmental factors that may contribute to it, and thus complicate its interpretation. We also present individual data from two 60-d head-down bed rest (HDBR) studies to evaluate the existence of IV, as HDBR is considered the most appropriate ground-based analog (Hargens and Vico, 2016) of cardiovascular and musculoskeletal deconditioning associated with spaceflight (Pavy-Le Traon et al., 2007).

## Evidence of IV in Human Spaceflight Data

**FIGURE 1:**
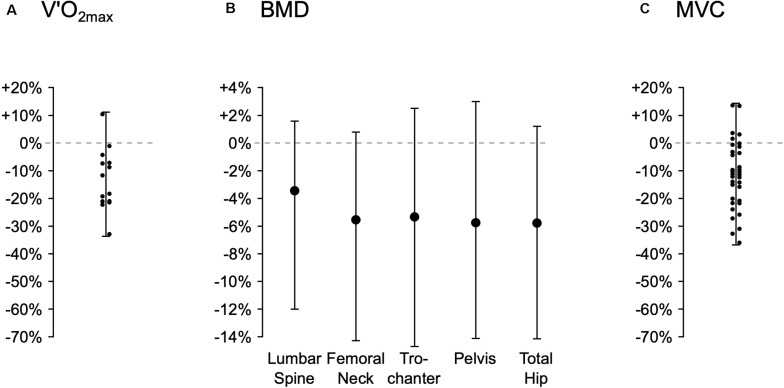
Range of individual changes (percentage change from pre- to post-flight) in key physiological parameters from spaceflight studies. The figure shows derived estimates of changes in maximal rate of oxygen uptake (VO_2max_, Panel **A**, extracted from Figure 3; Moore et al., 2014), bone mineral density (BMD, Panel **B**, extracted from Figure 2; Sibonga et al., 2015) and maximal force production of the knee extensors during a maximal voluntary contraction (MVC, Panel **C**, extracted from Figure 6; English et al., 2015). Minima, mean, and maxima data, and where possible (i.e., no overlap of data points) individual data points, were extracted from published figures using WebPlotDigitizer (United States). Thus, for Figure 2 of Sibonga et al. (2015), only the minima, mean and maxima data are presented.

A significant barrier in understanding IV in response to spaceflight is the manner in which data are published. The primary goal of most experiments in space is to compare data points (e.g., pre- to post-flight) or conditions/groups (e.g., crew following of two different diets) (Zwart et al., 2018) and, as such, data are typically presented only as group means with standard deviations/errors, although there are exceptions (Moore et al., 2014, Rittweger et al., 2018; McNamara et al., 2019). In addition to selected scientific experiments, a standard set of physiological measurements is performed before, during, and after flight as part of medical monitoring by the Space Agencies, and thus this data set comprises of data from astronauts that have flown on different space missions [e.g., NASA’s Shuttle and International Space Station (ISS)] with varying crew compositions and a wide range of durations (Smith et al., 2020). As these measurements are specifically for medical monitoring, they are not, by default, analyzed and published. However, some of these data have been published, both as group means and individual data (Spector et al., 2009; English et al., 2015; Sibonga et al., 2015). Data from spaceflight studies where individual data has been provided suggest that marked IV exists in the response of the musculoskeletal and cardiovascular systems during human space missions (Figure 1). However, the complex nature of human spaceflight missions means that this apparent IV must be interpreted with care.

## Interpretation of IV From Spaceflight Data

A major issue in understanding IV in response to the spaceflight environment – comprising of a number of factors such as microgravity, radiation, and space-specific nutrition – is the absence of astronauts who have performed no CM exercise (Scott et al., 2019). As many microgravity-induced physiological adaptations appeared to reflect those that occur with prolonged inactivity, physical exercise was identified as a key adaptation management strategy (Berry et al., 1962; Moore et al., 2010). As a consequence, CM exercise has been used in some form in almost all space missions. On ISS, exercise is the cornerstone of the CM program for long-duration missions (LDM) (Loehr et al., 2015) and Flight Rules dictate that all LDM crewmembers must perform CM exercise, which precludes abstinence, or intervention studies with a “no exercise” control group. As a result, all recent physiological data collected from space missions reflects the physiological responses to both the spaceflight environment *and* CM exercise.

Interpretation of spaceflight data with respect to IV is further complicated by the lack of standardization of the CM exercise program between agencies and individuals within a program, and thus can vary markedly between crewmembers (Rittweger et al., 2018). This is a result of several factors:

• Evolving CM exercise device technology. CM exercise devices have evolved over time and, in general, increased in their capacity to provide training stimuli. As a result, in the past, stronger, fitter crew may have been significantly limited by device capacity and thus received a sub-optimal exercise stimulus (Korth, 2015). It is possible that, only recently on ISS, have exercise programs not, in some way, been limited by device capacity. Even during the lifetime of ISS, the complement of exercise devices has changed, with more recent (post-2009-2010) crew having access to upgraded aerobic and resistance devices (Korth, 2015). As such, even when a study reports data from the same mission (e.g., ISS), if individual astronaut flights included in a study span a prolonged time period (e.g., English et al., 2015), it is possible that not all crewmembers had access to the same exercise devices.
• Increase in volume available for devices. With the advent of space “stations” (e.g., MIR and ISS), the volume available for exercise devices has increased and, as a result, so has the number and variety. As such, whereas previously crew had access to only a single, uni-modal (e.g., cycle ergometer) device, ISS crew are now able to perform both aerobic and resistance exercise, and choose between mechanically-loaded (treadmill running) and unloaded (cycle ergometry) aerobic exercise (Korth, 2015);
• Non-availability of devices within a mission. Sometimes exercise devices may not be available or their use constrained. For instance, on ISS between 2001 and 2004 on several occasions, one or more of the three exercise devices (CEVIS, T2, and iRED) were not operating nominally or unavailable (Hayes, 2015). As a result, even when a study reports data from crew who flew close to each other chronologically, it is possible that their use of devices differed;
• Operational factors and priorities. As important as CM exercise is considered by spaceflight operations, on occasions, it must be canceled due to activities related to arriving and departing vehicles, external “space walks,” science experiments and internal engineering/maintenance activities. Thus, through no fault of their own, an individual crewmember’s exercise program may be interrupted or constrained. In addition, both before and after missions, operational factors, and logistics can result in variation in the timing of pre- and post-flight measurements, and on occasions even cancellation at the request of the astronaut or attending Flight Surgeon.
• Adherence to the CM exercise program. Finally, astronauts’ attitudes to, and motivation for, exercise vary. Space Agencies provide crew with exercise devices, with exercise CM programs to follow, time to perform them, and education regarding their importance. However, the performance of CM exercise is an individual crewmember’s choice and thus engagement and adherence vary, including both the number, and intensity and duration, of sessions.

In summary, although the data in Figure 1 suggests marked IV in the biological response to the *combined* effects of the spaceflight environment and CM exercise, it is impossible to exclude the influence of factors including those outlined above. Indeed, some published data suggest that the quantity and “quality” of CM exercise may be a significant factor in the magnitude of spaceflight adaptation (Moore et al., 2014; Lee et al., 2015; Rittweger et al., 2018). However, as exercise performance metrics are considered private medical data by the Space Agencies, this data is rarely published or accounted for in the analysis.

There are also a number of individual biological effects related to exercise that may, in part, contribute to IV in spaceflight studies:

• Age. Astronauts have careers lasting several decades with the average age tending to increase (Smith et al., 2020). Age may influence bone’s adaptation to both mechanical loading (Rubin et al., 1992; Turner et al., 1995) and unloading (Perrien et al., 2007), as well as re-loading following unloading (Cunningham et al., 2018), although the effect upon the responsiveness of muscle (Cartee, 1994) or cardiorespiratory function (Mazzeo et al., 1984; Rossiter et al., 2005) is less clear. Thus, the importance of age across the typical astronaut career range (30–60 years old) is not well understood.
• Pre-flight physiological status. There is considerable variation between individual astronauts in the pre-flight values of parameters such as bone mineral density, muscle force production capacity, and maximal rate of oxygen uptake (VO_2__max_) (Orwoll et al., 2013; Moore et al., 2014; English et al., 2015; Sibonga et al., 2015). To what extent this reflects genetic differences and/or the effects of physical activity (i.e., adaptation) is unknown, but they may reflect differences in training history and thus training status. If so, the transition from Earth’s gravity (normal mechanical loading) to space (no mechanical loading), may represent markedly different adaptive stimuli for different crewmembers. This may also be true for the transition from pre-flight exercise habits – which are likely highly variable between crewmembers and may also vary within an individual in the intensive pre-launch period – to the high volume ISS CM exercise program (Korth, 2015), as prior capacity may influence the response to an aerobic exercise intervention (Milanović et al., 2015) and training history to a strength intervention (Mangine et al., 2018). Some bed rest data does suggest the reduction in VO_2__max_ is dependent on the initial level of aerobic fitness (Convertino, 1997) consistent with a greater magnitude of spaceflight adaptation (loss of aerobic fitness or muscle strength) in crewmembers who have higher pre-flight values (Moore et al., 2014; English et al., 2015).
• Responders vs. non-responders. Differences in pre-study status may, in part, explain the apparent IV in response to terrestrial exercise interventions. Terrestrial studies demonstrate IV in post-exercise training adaptations, with some subjects exhibiting no meaningful improvements (Bouchard and Rankinen, 2001; Timmons, 2010) or even a decrease in capacity (Bouchard et al., 2012). Recent evidence (Pickering and Kiely, 2019), however, suggests that it is unlikely that global non-responders to exercise exist, that the “non-response” can be mitigated by changes in training variables, and that individual responses to an intervention should be considered specific to that intervention, at that time, and with the selected outcome measures. Due to the high number of factors that might influence the pre- to post-flight change in the physiological variables measured from astronauts, to what extent this is the case with the performance of in-flight CM exercise is difficult to elucidate.

Spaceflight also exposes crewmembers to several unique factors that may contribute to spaceflight-induced adaptation and the response to CMs. Radiation exposure in space is markedly different compared to that on Earth, which is associated with a range of biological effects that can differ between tissues and systems (Chancellor et al., 2014). Moreover, radiation may also influence the effects of microgravity (Yatagai et al., 2019). As such, combined with the fact that there is significant IV in the sensitivity to radiation (Cucinotta, 2001), radiation exposure could be a contributor to astronaut physiological IV. Furthermore, although ISS crew are limited to the (largely pre-packaged) on-board food supply and receive nutritional guidance on the optimal quantity and combination of foodstuffs to consume, they are free to choose their own food from the pantry. As a result, nutritional intake varies between individuals and, possibly combined with space-specific issues such as motion sickness, loss of appetite, and difficulties in metabolizing food in microgravity (Laurens et al., 2019), may result in different energy intakes. Energy intake required for energy balance varies with body size and the level of physical activity (including CM exercise) (Scott et al., 2020) and there is evidence of a negative energy balance (Stein, 2000) and the loss of body mass (Wade et al., 2002; Matsumoto et al., 2011) in-flight. Indeed, CM exercise itself may be a key factor in generating this imbalance (Laurens et al., 2019). The loss of body mass in space is associated with decreased muscle mass and functionality, incidence of cardiovascular issues, and even oxidative stress (Stein, 2002; Smith and Zwart, 2008). Terrestrial studies of an energy deficit demonstrate comparable effects (Bergouignan et al., 2016), whilst the deleterious consequences of an energy deficit on health, and the adaptive response to physical activity are well documented (Ihle and Loucks, 2004; Bergouignan et al., 2016; Murphy and Koehler, 2020), including in both athletic (Sale and Elliott-Sale, 2019) and military (Murphy et al., 2018) populations. Thus, in-flight energy balance differences (Bergouignan et al., 2016) may contribute to IV in the physiological adaptive responses to spaceflight. Finally, crewmembers complete a fluid loading protocol in the hours before landing to reduce the risk of orthostatic intolerance (Bungo et al., 1985) and may also be administered a saline infusion on landing, the volume of which is determined by medical personnel as clinically indicated. The effects of both of these treatments (if administered and in what quantity) may have individual effects on fluid volumes and associated cardiovascular function and performance.

Even if all of the factors described above could be controlled or eliminated, an additional consideration is to what extent the observed IV is “true” biological IV in response to the spaceflight environment. Atkinson and Batterham (2015) argue that, because both technical error and random within-subject variation are inherent within any measurement, IV cannot be confirmed from the pre- and post-intervention (or exposure) measurements alone. Thus, without an appropriate control group, there is a risk of identifying “false” IV (Williamson et al., 2017). Published data from spaceflight do not include a control group, either one that performs no exercise (to investigate the effects of CM exercise), or a ground-based group (to compare to those in space). Thus, confirmation of the presence of “true” IV is not possible, either in response to the spaceflight environment or the use of CM exercise.

## Role of Head-Down Bed Rest in Understanding IV in Spaceflight

Long-term HDBR is the pre-eminent ground-based experimental approach to study the effects of prolonged gravitational unloading and disuse (Hargens and Vico, 2016). Like all Earth-based analogs, HDBR is confounded by the presence of gravity and the absence of space radiation exposure rendering it unsuitable as a model for spaceflight-induced adaptation in all physiological systems. Specifically in relation to the presence of gravity, unlike spaceflight, HDBR may not affect signaling from the semicircular canals or the otoliths (Dupui et al., 1992), with only the somatosensory system being affected. This may result in differential effects in outcome measures that directly test these systems (e.g., postural stability), those that may be influenced by them, such as blood pressure (Hallgren et al., 2015), and performance in functional tasks to which they may contribute (Miller et al., 2018; Mulavara et al., 2018). Despite these limitations, however, HDBR is still considered a valid analog for the musculoskeletal and cardiovascular systems (Pavy-Le Traon et al., 2007) and, as such, may be a valuable tool for the determination of the presence of, and factors determining, IV in the response to gravitational unloading and performance of CMs. Although the typical duration of current ISS LDMs (∼6-months) exceeds the duration of even the longest HDBR studies (90-d) and it remains unknown if the duration of exposure to microgravity or axial unloading leads to increased or decreased IV, HDBR is free of many of the factors that potentially confound the interpretation of spaceflight data:

• The majority of studies include a group of subjects whom are exposed *only* to HDBR. Where the primary goal is to test a CM, this group serves as the control group against which the CM is compared, allowing the effects of HDBR alone to be isolated. The inclusion of a control group has the added advantage of allowing “true” IV in response to the CM (but not HDBR alone as there is no ambulatory control condition) to be detected by comparing the standard deviations of the two groups (Atkinson and Batterham, 2015)];
• When a CM is applied, it is applied in a systematic and rigorous manner, and any deviations accurately recorded.
• Experimental conditions are tightly controlled, thus reducing the potential impact of “non-exercise” biological factors (e.g., nutrition) and eliminating the “operational” factors (e.g., vehicle visits, spacewalks, and engineering) present in spaceflight.

**FIGURE 2:**
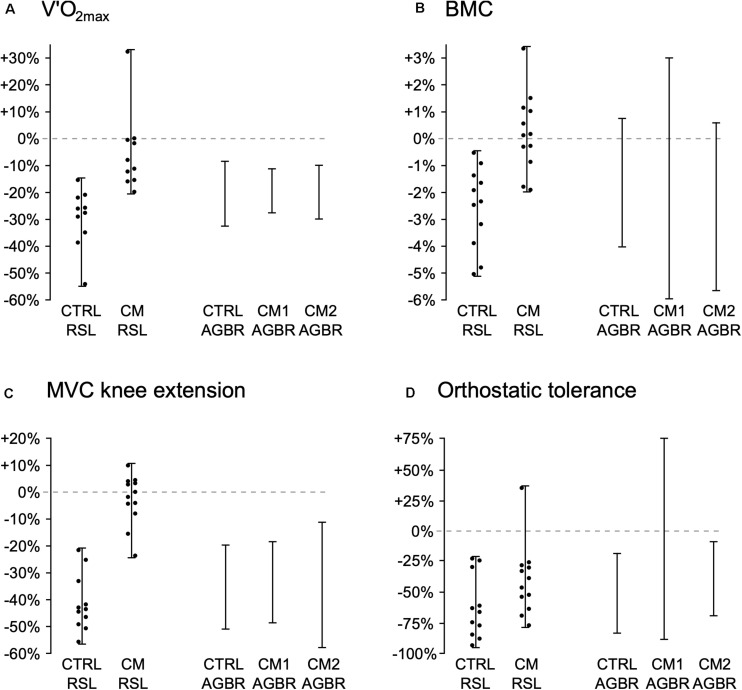
Range of individual changes (percentage change from pre- to post-study) in the control (CTRL) and countermeasure (CM) groups from two European Space Agency (ESA) 60-d, -6° head-down bed rest (HDBR) studies: ESA’s “*Reactive jumps in a Sledge jump system as countermeasure during Long-term bed rest*” (RSL) study, in which HDBR only (CTRL RSL) was compared to 48 training sessions of “reactive jumps,” consisting of 4 × 12 countermovement jumps and 2 × 15 repetitive hops (CM RSL; see Kramer et al., 2017a for full description of protocol) and from ESA’s “*Artificial Gravity Bed Rest – European Space Agency*” (AGBRESA) study, in which HDBR only (CTRL AGBR) was compared to two different artificial gravity protocols, 1 × 30-min session/day of supine centrifugation at +1Gz at the center of mass (AGBR CM1; see Laing et al., 2020 for full description of protocol), and 6 × 5-min/day (all separated by 5-min rest breaks) supine centrifugation at +1Gz at the center of mass (AGBR CM2; see Laing et al., 2020). The figure shows changes in maximal rate of oxygen uptake (VO_2__max_, Panel **A**), bone mineral content (BMC) as assessed by pQCT of the tibia at 98% of tibial length (Panel **B**), force production during a maximal voluntary contraction (MVC) of the knee extensors (Panel **C**), and orthostatic tolerance (OT) time during a tilt-table test (Panel **D**). Individual values from the RSL study come from the data (mean and standard deviations) sets published in Kramer et al. (2017b), where the full study protocol is also described, but individual values were not published. The range of individual values (individual maximum and minimum changes) from the AGBRESA study was provided courtesy of ESA’s Human Research Office. Individual values from the AGBRESA study could not be provided as these data have not yet been published and will be included in future publications assessing the efficacy of the artificial gravity countermeasures.

However, despite the potential value of HDBR in understanding IV in the response to gravitational unloading and CMs, as the majority of published HDBR data are from science experiments (most typically comparing the CM and control groups), there is again a scarcity of individual data from which to assess IV in response to HDBR alone and to CMs. Figure 2 shows individual data from two recent European Space Agency (ESA) 60-d HDBR studies, the “*Reactive jumps in a Sledge jump system as countermeasure during Long-term bed rest*” (RSL) and “*Artificial Gravity Bed Rest – European Space Agency*” (AGBRESA) studies, both including control and CM (RSL: reactive jumps; AGBRESA: artificial gravity) groups. These data suggest that, despite the high degree of standardization, and the control of many (but not all) of the factors that could influence the response to the spaceflight environment and CM exercise, there appears to be substantial IV in both the cardiorespiratory and musculoskeletal response to HDBR, and in each of the three different CM regimes. Specifically, after 60-d of HDBR *alone*, individual changes in VO_2__max_, tibial bone mineral content as assessed by pQCT, knee extensor maximal force production, and orthostatic tolerance, ranged from −54% to −9%, −5% to +1%, −56% to −20%, and −94% to −19%. Changes with the RSL’s jumping intervention for these outcome measures ranged from −20% to +32%, −2% to +4%, −24% to +10%, and −78% to +35%, and with the AGBRESA’s artificial gravity interventions from −29% to −11%, −6% to +3%, −58% to −9% and −87% to +76%.

A further advantage of the HDBR model in investigating IV is the attempt to standardize conditions between studies. This standardization has resulted in two distinct, but equally important outcomes: standardization of conditions between studies and a standard set of “core” measurements from every study (Sundblad et al., 2016). As a result of their complexity and expense, bed rest studies are, and will likely continue to be, small, with typically only 8–12 subjects in each group. However, standardization of conditions and outcome measures between studies means that, in principle, results of different studies, in particular the control groups, can now be not only compared, but also potentially combined. Specifically, in relation to IV, data from comparable studies (e.g., 60-d of -6° HDBR only) could be pooled. Thus, the more studies that adhere to the standardization guidelines, the larger this pool will become.

## Discussion

Whilst the spaceflight data presented in this Perspective suggests a marked degree of IV in response to long-term spaceflight, it is clear that numerous biological, operational, and environmental factors may contribute to this, and thus complicate its interpretation. As such, we conclude that it is currently not possible to evaluate IV in response to the spaceflight environment, and/or the use of CM exercise. In contrast, despite highly standardized experimental conditions, IV is also evident in response to long-duration HDBR. Thus, we propose that HDBR is suitable for the investigation of IV in the physiological response to gravitational unloading and CMs. Such analysis could represent the first critical step in understanding the existence of IV in spaceflight adaptation. Should “true” IV be confirmed, investigation of possible mediators will be warranted. In turn, this may provide insights into the potential role of IV in the apparent effectiveness of current and future CMs. In the longer-term, characterization of IV may even aid the selection of individuals for specific exploration missions, where a comprehensive understanding of the effects of the spaceflight environment and the effectiveness of CMs will be critical to the successful execution of mission objectives and the safe return of crews to Earth.

## Data Availability Statement

The data analyzed in this study is subject to the following licenses/restrictions: Data from the European Space Agency (ESA)’s “AGBRESA” study are currently owned by ESA and were provided courtesy of ESA’s Human Research Office. As such, they are not currently publicly available. The re-analysis of spaceflight data was performed directly from previously published figures and we do not know if the datasets are publicly available. Data from the ESA “RSL” study are owned by the authors and can be made available. Requests to access these datasets should be directed to AK, andreas.kramer@uni-konstanz.de.

## Ethics Statement

The studies involving human participants were reviewed and approved by the Ethics Committee of the Northern Rhine Medical Association (Ärztekammer Nordrhein) in Duesseldorf, Germany, and Federal Office for Radiation Protection (Bundesamt für Strahlenschutz). The patients/participants provided their written informed consent to participate in these studies.

## Author Contributions

JS wrote the first draft of the manuscript. All authors commented on previous versions of the manuscript, and read and approved the final manuscript.

## Conflict of Interest

JS, NP, and DG are all employed by KBR GmbH, Cologne, Germany. The remaining author declares that the research was conducted in the absence of any commercial or financial relationships that could be construed as a potential conflict of interest.
